# Rh_2_(CF_3_CONH)_4_: The First Biological Assays of a Rhodium (II) Amidate

**DOI:** 10.1155/MBD.1997.333

**Published:** 1997

**Authors:** Breno Pannia Espósito, Szulim Ber Zyngier, Aparecido Ribeiro de Souza, Renato Najjar

**Affiliations:** 1 Institute of Chemistry University of São Paulo Av. Lineu Prestes 748 São Paulo 05508-900 Brazil; 2 Institute of Biomedical Sciences - I University of São Paulo Av. Lineu Prestes 1524 São Paulo 05508-900 Brazil; 3 Institute of Chemistry Federal University of Goiás Campus II P.O. Box 131 Goiânia 74001-970 Brazil

## Abstract

The rhodium (II) complexes Rh_2_(tfa)_4_.2(tfac) and Rh_2_(tfacam)_4_ (tfacam = CF_3_CONH-,tfa = CF_3_COO-,tfac = CF_3_CONH_2_) were synthesized and characterized by microanalysis and electronic and vibrational spectroscopies. Rh_2_(tfacam)_4_ was tested both *in vitro* (U937 and K562 human leukemia cells and Ehrlich ascitic tumor cells) and *in vivo* for cytostatic activity and lethal dose determination, respectively. This is the first rhodium tetra-amidate to have its biological activity evaluated. The LD_50_ value for Rh_2_(tfacam)_4_ is of the same order as that of cisplatin, and it was verified that the rhodium complex usually needs lower doses than cisplatin to promote the same inhibitory effects.


### $Rh_2(CF_3CONH)_4$: THE FIRST BIOLOGICAL ASSAYS OF A RHODIUM (II) AMIDATE

Breno Pannia Espósito[1], Szulim Ber Zyngier[2], Aparecido Ribeiro de Souza[3] and Renato Najjar[1]

[1] Institute of Chemistry, University of São Paulo, Av. Lineu Prestes 748, 05508-900, São Paulo, Brazil <bpesposi@quim.iq.usp.br>,
[2] Institute of Biomedical Sciences - I, University of São Paulo, Av. Lineu Prestes 1524, 05508-900, São Paulo, Brazil;
[3] Institute of Chemistry, Federal University of Goiás, Campus II, P.O. Box 131, 74001-970, Goiânia, Brazil.

**ABSTRACT**

The rhodium (II) complexes $Rh_2(tfa)_4.2(tfac)$ and $Rh_2(tfacam)_4$ (tfacam = $CF_3CONH^-$,tfa = $CF_3COO^-$, tfac = $CF_3CONH_2$) were synthesized and characterized by microanalysis and electronic and vibrational spectroscopies. $Rh_2(tfacam)_4$ was tested both *in vitro* (U937 and K562 human leukemia cells and Ehrlich ascitic tumor cells) and *in vivo* for cytostatic activity and lethal dose determination, respectively. This is the first rhodium tetra-amidate to have its biological activity evaluated. The $LD_{50}$ value for $Rh_2(tfacam)_4$ is of the same order as that of cisplatin, and it was verified that the rhodium complex usually needs lower doses than cisplatin to promote the same inhibitory effects.

**INTRODUCTION**

In 1969, Rosenberg and coworkers studied the effect of electric fields on the growth of *Escherichia coli* and noted that platinum compounds of *cis* configuration showed cytostatic activity [1]. Today, platinic derivatives are widely used in tumor therapy, specially against testicular or ovarian carcinomas, and bladder, head and neck tumors [2]. The biological activity of cisplatin and other platinum derivatives has already been the scope of numerous reviews [3].

Figure 1

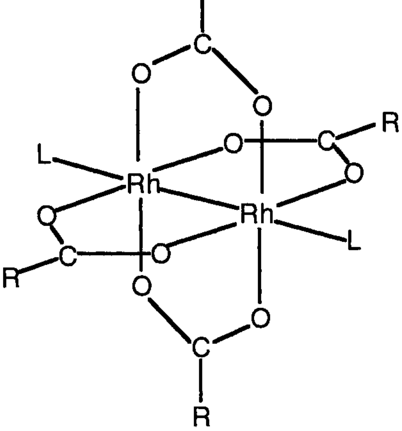

Figure 2

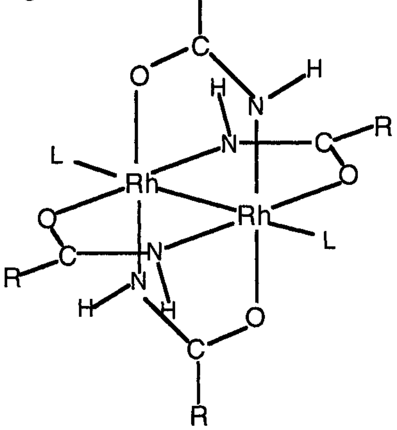

Following the pioneering work of Rosenberg, complexes of transition metals other than platinum such as titanium, ruthenium [2], iridium [4] and rhodium have been and are still being tested for antitumor activity. The rhodium carboxylates, whose dimeric structure is shown in Figure 1 (R = bridging ligand substituent group; L = axial coordinating ligand which may be absent), are particularly noteworthy among the rhodium compounds.

The antitumor activity of rhodium complexes was first reported by Bear and coworkers [5]. Rhodium butyrate (R = $C_3H_7$ in Figure 1) showed high *in vitro* cytostatic potential and *in vivo* antitumoral activity but with toxicity higher than that of cisplatin [2,6,7]. For this reason, this class of dinuclear complexes remains under study, and it is expected that structures may be found that ideally would have maximum antineoplasic effect with minimum or no toxic effect. Several dinuclear rhodium (II) complexes have been synthesized with this objective [8].

The hopes regarding the chemotherapeutic potential of rhodium amidates (Figure 2) are based on the pioneering work described by Bear [9] and on previous findings for platinum and other metal compounds

[3a,10] which show that one of the main factors determining biological activity is the presence of at least one free NH group in the molecule in order to establish hydrogen bonds with nucleotide phosphate groups.

A number of rhodium tetra-amidates have been synthesized and characterized [11] and in this work we report some chemical properties and the pharmacological parameters $IC_{50}$ and $LD_{50}$ of the complex, $Rh_2(tfacam)_4$ (Figure 2; R = $CF_3$); L can be a water molecule. To our knowledge, this is the first time that a rhodium tetra-amidate has had its biological activities reported [12].

### MATERIALS AND METHODS

***Reagents***: $RhCl_3.xH_2O$ was purchased from Fluka. Chloroform and trifluoroacetamide (tfac) were from Aldrich Chemicals. A commercial solution of cisplatin (*Platinil 10*) was from Quiral Química do Brasil S/A and Tween™-80 was from Baker. Using previously described methods, we obtained rhodium acetate, $Rh_2(ac)_4$ [13]. Rhodium trifluoroacetate (R = $CF_3$ and L is absent in Figure 1) was obtained in yields of ~ 60% through dropwise addition of ethanolic solutions of $RhCl_3$ over $NaOOCCF_3$, a slight modification of a method described earlier [8c].

***Melting reaction***: The synthesis of the complex $Rh_2(CF_3CONH)_4$ was adapted from the literature [11a,b]. The starting reagents $Rh_2(ac)_4$ and tfac were previously treated in order to achieve the desired reaction course. The amide (~ 2 g; 17.7 mmol) was dried under vacuum for 30 minutes, at temperatures between 20 and 40°C, to eliminate any trace of water or trifluoroacetic acid. 200 mg (0.45 mmol) of $Rh_2(ac)_4$ were heated to 100°C for 30 minutes in a stoppered 50 mL round flask with a magnetic stirrer bar. This system was immersed in a 144 to 148°C silicon oil bath for 2 hours. The flask was sealed with Teflon™ tape after inner pressure compensation, which occurred after some seconds.

After cooling, the crystals were sublimed during 10 hours in an Abderhalden equipment heated to 56°C with acetone. An amorphous and hygroscopic purple solid was obtained whose elemental analysis was consistent with the stoichiometry $Rh_2(CF_3CONH)_4(CF_3CONH_2)_2$ (Calculated: C = 16.38%; H = 0.59%; N = 9.55%; Experimental: C = 16.12%; H = 1.59%; N = 9.27%).

***$Rh_2(tfa)_4.2(tfac)$*** (L = $CF_3CONH_2$ in Figure 1): The adduct between $Rh_2(tfa)_4$ and tfac was prepared by refluxing the reagents in chloroform for 10 minutes, followed by slow solvent evaporation in air. (Calculated: C = 16.31%; H = 0.46%; N = 3.17%; Experimental: C = 16.23%; H = 0.63%; N = 2.91%).

***Electronic Spectroscopy***: The electronic spectra in the range of 190 to 700 nm were registered with a Hitachi U-3000 Spectrophotometer, with 1 cm quartz cells using W and $D_2$ lamps. The aqueous solutions were $10^{-3}$ and $10^{-4}$ M for visible and ultraviolet (UV) spectrometry, respectively.

***Vibrational Spectroscopy***: The infrared spectra in the range of 500 to 4000 $cm^{-1}$ were obtained with a Perkin-Elmer FT-IR equipment, in KBr pellets. The Raman spectra of the rhodium complexes were obtained in a Renishaw Ramascope equipment, with $Ar^+$ (488 nm; $Rh_2(tfa)_4.2(tfac)$) or He/Ne (633 nm; $Rh_2(tfacam)_4.2(tfac)$) laser excitation.

***In vitro Biological Assays***: Cisplatin $1x10^{-3}$ M and $Rh_2(tfacam)_4$ 0.8 M aqueous solutions were prepared. U937 and K562 human leukemia cells were cultivated in RPMI-1640 medium and kept in tissue culture flasks at 37°C. Ehrlich ascitic cells were kept in Balb-c mice, sacrificed at the time of experiment, and from which 1 mL of intraperitonial liquid was extracted.

Inside a laminar flow cabinet, these cells were transferred to centrifuge tubes that were sealed and centrifuged at 1400 rpm for 5 minutes. The cell pellet was resuspended in a mixture of culture medium and PBS buffer solution. Cell counting was performed in Neubauer plates, working typically in the range of 3 to 4 x $10^5$ cells/mL.

In the wells of tissue culture plates 1 mL of cell solution, variable volumes of drug solutions and sufficient saline to equalize the dilutions were then added. The culture plates were incubated for 24 hours at 37°C with 5% $CO_2$ atmosphere. Control cells received only saline.

After 24 hours, the content of each well was pipetted to Eppendorf flasks and centrifuged at 1500 rpm for 3 minutes. The supernatants were discarded and the pellet was resuspended in 500 mL of PBS and 500 mL of Trypan blue solution. A small volume of each flask was transferred to Neubauer plates and the living and dead cells were counted.

***In vivo Biological Assays***: After preliminary tests, a $2.5x10^{-3}$ M aqueous solution of $Rh_2(tfacam)_4$ containing 5% w/w of Tween™-80 was prepared, in order to achieve adequate solubilization of the inorganic compound. This solution was prepared by dissolving 0.071 g (0.079 mmol) of $Rh_2(tfacam)_4.2(tfac)$ in the minimum possible amount of an acetone-ethanol mixture (~ 10:1) in a 25 mL flask, forming a deep red solution characteristic of axial coordination with acetone. Then 1.25 g of Tween™-80 was added, followed by manual homogeneization and elimination of the organic solvents through $N_2$ bubbling for 1 to 1.5 hours. After evaporation, distilled water was carefully added to complete 25 mL. This final solution was purple.

Volumes of this solution ranging from 0.2 to 0.5 mL were injected intraperitoneally in groups of 10 to 11 male Balb-c mice (average weight: 22.5 g). The dead animals were counted after 6 days.

### RESULTS AND DISCUSSION

***Melting Reaction***: The yield of the reaction between $Rh_2(ac)_4$ and tfac was quantitative and the described method is reproducible. Drying of the amide proved to be important since traces of trifluoroacetic acid, the tfac hydrolysis product, could be involved in reduction processes of Rh(II) to $Rh^0$, a phenomenon observed during our early tests.

$Rh_2(tfacam)_4$ is insoluble in chloroform, soluble in acetone, methanol and ethanol and moderately soluble in water. Under prolonged heating its solubility decreases significantly, a behavior similar to some Rh binuclear compounds such as citrate and other alkanoates [14]. In such situations, the molecules probably undergo polymerization, in which some of the oxygen atoms of the bridging ligands coordinate at the axial position of a neighboring molecule forming chains.

Methanol solutions of $Rh_2(tfacam)_4.2(tfac)$ were prepared and mixed with an excess of several ligands that could, in principle, displace the tfac molecules and occupy the axial positions. The aim in this case was a qualitative verification of the chemical properties of this complex. Table I reports the results. These data show that the axial positions are passive to ligand exchange, giving rise to new adducts with color patterns different from that of Rh carboxylates. These usually show blue or green adducts for coordination through O, pink or red for coordination through N and orange through S or P. In the case of $Rh_2(tfacam)_4$, adducts coordinated axially through O are reddish or purple and are yellow for coordination with pyridine, DMSO or $R_3P$, reflecting changes in the energy levels of the molecular orbitals of the compounds [11a,e].

In crystals, it was observed [11b] that the $Rh_2(tfacam)_4$ complex can display hydrogen bonds between the amide H atom and one of the F atoms of the $CF_3$ group, which would be approximately in the NCO plane (Figure 3). Whether or not this interaction is more important than that of NH groups with proteic substrates in biologic environments is an issue for future research.

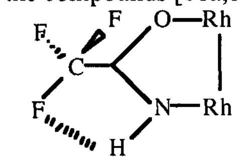

Figure 3

For the tetra-amidates there are four possible geometric isomers, according to the position of the NH groups. Generally, the isomer with Rh-N bonds in *cis*, as in Figure 2, is preferentialy obtained (~ 94% [11b,c]), a fact that led Bear and coworkers to suggest the restriction that "the nitrogen of the entering amidate ion cannot bind *trans* to any other nitrogen on the same rhodium atom" [11d].

***Electronic Spectroscopy***: Table II summarizes the visible and UV spectral data in aqueous solutions. The electronic transitions of rhodium carboxylates have already been the scope of a number of reports [15a-c], with the assignements shown in Table II being widely accepted.

The electronic spectra of rhodium carboxylates and tetra-amidates (as $Rh_2(CH_3CONH)_4$ and $Rh_2(CF_3CONH)_4$) are qualitatively similar [15d], although rhodium amidates possess a band at ~ 350 - 400 nm which sometimes cannot be observed, due to an overlap with UV absorption, and was tentatively assigned to a $\pi^*_{Rh-Rh}$ to $\sigma^*_{Rh-O}$ or $\sigma^*_{Rh-N}$ transition [11b].

Table I: Effect of ligand addition to $Rh_2(tfacam)_4.2(tfac)$

| Ligand | $\lambda_1$*/ nm |
|---|---|
| Pyridine | 472 |
| Dimethylsulfoxide (DMSO) | 482 |
| Imidazole | 480 (shoulder) |
| Acetonitrile | 508 |
| Dimethylformamide | 540 |

* $\lambda_1$ is a typical absorption band of the Rh dimeric compounds (~500-600 nm). Its position is altered by axial substitution and defines the apparent color of the adducts.

Table II: $\lambda$ (nm) and $\varepsilon$ ($M^{-1}cm^{-1}$) for some rhodium complexes in water solution

| Complex | $\lambda_1$ | $\varepsilon_1$ | $\lambda_2$ | $\varepsilon_2$ | $\lambda_3$ | $\varepsilon_3$ | $\lambda_4$ | $\varepsilon_4$ |
|---|---|---|---|---|---|---|---|---|
| $Rh_2(tfa)_4$ | 581 | 169 | 442 | 115 | 255(s) | ~4400 | 226 | 13000 |
| $Rh_2(tfacam)_4$ | 552 | 43.9 | — | — | ~250(s) | — | 218(s) | ~7000 |

$\lambda_1$: $\pi^*_{Rh-Rh}\rightarrow\sigma^*_{Rh-Rh}$; $\lambda_2$ : $\pi^*_{Rh-Rh}\rightarrow\sigma^*_{Rh-O}$; $\lambda_3$ (shoulder): $\sigma_{Rh-Rh}\rightarrow\sigma^*_{Rh-Rh}$; $\lambda_4$ : $\sigma_{Rh-O}\rightarrow\sigma^*_{Rh-Rh}$

(s) shoulder —: not observed

***Vibrational Spectroscopy***: In KBr pellets, primary amides have several characteristic infrared (IR) frequencies: ($_{NH,as}$ at ~ 3330 and ($_{NH,s}$ at ~ 3180 $cm^{-1}$; a band at ~ 1650 $cm^{-1}$ corresponding to ($_{CO}$ (*Amide I* band); a $\delta_{NH}$ (*Amide II* band) at 1515 - 1650 $cm^{-1}$ and weak one at ~ 1400 $cm^{-1}$ from ($_{CN}$. Amide I and Amide II bands may sometimes overlap. ($_{CF}$ usually falls in the 1000-1400 $cm^{-1}$ range [16]. Table III summarizes the relevant IR data for the free ligand tfac and some rhodium complexes. Table IV lists the main bands observed in the 100 to 500 $cm^{-1}$ range. The Rh-Rh bond stretching frequency is practically independent of substitutions in the bridge ligands, but depends strongly on the nature and influence of the axial ligands. Its assignment has already been the subject of some controversy [cfr 17-21]. Using normal coordinate analysis, Pruchnik and coworkers [17] concluded that both of the reported bands (in the ~ 170 -

180 and in the ~ 280 - 300 cm$^{-1}$ region) show a component of the Rh-Rh vibration, not as pure bands but instead coupled with Rh-$O_{bridge}$ vibrations, for example.

Table III: Main IR bands for the complexes (cm$^{-1}$), in KBr pellets

| Compound | Bands / cm$^{-1}$ | Proposed assignment |
|---|---|---|
| tfac(freeligand) | 3370 (s) | $\nu_{NH,as}$ |
| | 3192 (s) | $\nu_{NH,s}$ |
| | 1644 (broad) | overlap Amide I/Amide II |
| | 1458 (m) | $\nu_{CN}$ |
| | 1153 (s) | $\nu_{CF}$ |
| $Rh_2(tfa)_4$ | 1668 (s) | $\nu_{CO,as}$ |
| | 1461 (w) | $\nu_{CO,s}$ |
| | 1194 (s) | $\nu_{CF}$ |
| $Rh_2(tfacam)_4$.2(tfac), see Figure 6 | 3330 (broad) | $\nu_{NH}$ (overlap as/s) |
| | 1659 (s) | overlap AmideI/Amide II |
| | 1431 (m) | $\nu_{CN}$ |
| | ~1178(s) | $\nu_{CF}$ |
| $Rh_2(tfa)_4$.2(tfac) | 3446(s) | $\nu_{NH}$ (overlap as/s) |
| | 1663(s) | (Am.I/Am.II) or $\nu_{CO}$,carbox [1] ? |
| | 1459(m) | $\nu_{CO,s}$ or $\nu_{CN}$ ? |
| | 1192(s) | $\nu_{CF}$ |

[1]: $\nu_{CO,carbox}$ = CO stretching from rhodium carboxylate
s = strong; m = medium; w = weak

With amidates as bridging ligands, the Rh-Rh bond will be subject to a different environment. $Rh_2(CH_3CONH)_4$ or $Rh_2(CF_3CONH)_4$ have Rh-Rh distances about 0.4 nm greater and $\nu_{Rh\text{-}Rh}$ around 15 cm$^{-1}$ [23] smaller than $Rh_2(ac)_4$. These effects could be explained either by steric reasons (the amidate O-C-N angles are slightly bigger than the O-C-O angle of the carboxylates) or by electronic reasons (amidates are better $\pi$-acceptors than carboxylates, and so they can remove electron density from the Rh-Rh bond) [15,22].

A tentative assignment of the Rh-Rh vibration was carried out for the compounds $Rh_2(tfa)_4$.2(tfac) and $Rh_2(tfacam)_4$.2(tfac). The $\nu_{Rh\text{-}Rh}$ value for $Rh_2(tfa)_4$ was reported as being 332 cm$^{-1}$ [8h]. Axial coordination inserts electrons in antibonding levels of the Rh-Rh bonding molecular orbitals, so we would expect a slight weakening of this bonding and a consequent decrease of the $\nu_{Rh\text{-}Rh}$ value when comparing $Rh_2(tfa)_4$ with $Rh_2(tfa)_4$.2(tfac). This is indeed observed if we consider the predominant Rh-Rh vibrational band as being that at 327 cm$^{-1}$ in Table IV. There is also a trend towards weakening the Rh-Rh bond if one compares $Rh_2(tfa)_4$.2(tfac) with $Rh_2(tfacam)_4$.2(tfac). This is in fact observed if we consider the band at 313 cm$^{-1}$ in the Raman spectrum for the complex $Rh_2(tfacam)_4$.2(tfac) (Table IV) to be mainly Rh-Rh in character.

Table IV: Main Raman bands for the complexes

| Complex | Absorption / cm$^{-1}$ |
|---|---|
| $Rh_2(tfa)_4$.2(tfac) | 157 (s); 192 (m); 282 (w); 327 (m); 464 (w); 541 (w) |
| $Rh_2(tfacam)_4$.2(tfac) | 173 (w); 179 (w); 313 (s); 432 (w); 502 (w); 524 (m) |

***In vitro Biological Assays***: After counting the live and dead cells of the different lines exposed to the two tested drugs, we built the suitable dose-response curves using *probit transformation*, a statistical treatment that changes values of a normal curve into a straight line. The $IC_{50}$ parameter was determined from all these curves and the final values are presented in Table V. Both in this case and in the $LD_{50}$ determinations, all values reported were calculated under the assumption that the axial L positions were occupied with water molecules after solubilization of $Rh_2(tfacam)_4$.

Table V: Average $IC_{50}$ (x10$^{-5}$M) values of the assayed drugs

| Tumor lineage | $Rh_2(tfacam)_4$ | Cisplatin |
|---|---|---|
| U937 | 4.8 | 13.0 |
| K562 | 7.8 | 7.9 |
| Ehrlich | 3.0 | 6.4 |

Table VI: $LD_{50}$ values

| Dose (mL) | Live/Dead | Total of animals | Conc. (mol/kg) |
|---|---|---|---|
| 0.2 | 8/2 | 10 | $2.2 \times 10^{-5}$ |
| 0.3 | 10/1 | 11 | $3.4 \times 10^{-5}$ |
| 0.4 | 8/3 | 11 | $4.5 \times 10^{-5}$ |
| 0.5 | 1/9 | 10 | $5.6 \times 10^{-5}$ |

Analysing the comparative data of $IC_{50}$ we see that the rhodium drug usually requires half of the molar dose of cisplatin to achieve the same inhibitory effect. Nevertheless, with respect to K562 cells, both act in the same concentration ranges.

***In vivo Biological Assays***: Table VI shows the results of $LD_{50}$ determinations employing male Balb-c mice.

We obtained $LD_{50}$ of the rhodium complex as being $4.8 \times 10^{-5}$ mol/kg (fidutial limits: $6.1 \times 10^{-5}$ and $3.7 \times 10^{-5}$ mol/kg). $LD_{10}$, the lethal dose for 10% of the population, is a good first approach for tumor therapy tests. For $Rh_2(tfacam)_4$, its value is $2.4 \times 10^{-5}$ mol/kg (fidutial limits: $3.7 \times 10^{-5}$ and $1.5 \times 10^{-5}$ mol/kg). In their review, Hydes and Russell [23a] reported the $LD_{50}$ of intraperitoneally administered cisplatin in Swiss white female mice [23b] as being $4.33 \times 10^{-5}$ mol/kg. Assuming that this value can be compared with our results, we see that $Rh_2(tfacam)_4$ has the same order of toxicity as cisplatin.

### CONCLUDING REMARKS

These findings encourage us to continue research with the $Rh_2(tfacam)_4$ complex. Presentely, we are undertaking survival rate determinations and anatomopathological experiments in Balb-c mice, in order to explore the potential of our compounds in cancer treatment. Another line of research will involve the interaction of this complex with selected proteins.

### ACKNOWLEDGEMENTS

The authors are grateful to Fundação de Amparo à Pesquisa do Estado de São Paulo (FAPESP) and to Conselho Nacional de Desenvolvimento Científico e Tecnológico (CNPq) for financial support. We would like to thank Profª Márcia Temperini from the Molecular Spectroscopy Laboratory (LEM-IQUSP) for the Raman spectra. One of the authors (BPE) is also grateful to the CNPq for a MSc fellowship grant.

### REFERENCES

[1] a) Rosenberg, B., Van Camp, L. *Nature* 1969, **222**, 385 b) Rosenberg, B., Van Camp, L. *Cancer Res.* 1970, **304**, 1799 c) Rosenberg, B. *Cancer Chemother. Rep.* (1), 1975, **59**, 589 d) Rosenberg, B. *Interdisc. Sci. Rev.* 1978, **3**, 134 e) Rosenberg, B. *Biochemie* 1978, **60**, 859 f) Kociba, R.J.; Sleight, S.; Rosenberg, B. *Cancer Chemother. Rep.* (1), 1970, **54**, 325

[2] Keppler, B.K. *New J. Chem.* 1990, **14**, 389

[3] a) Reedijk, J *Pure & Appl. Chem.* 1987, **59(2)**, 181 b) Umapathy, P. *Coord. Chem. Rev.* 1989, **95**, 129 c) Zamble, D.B.; Lippard, S.J. *Trends in Biochem. Sci.* 1995, **20(10)**, 435 d) Wang, K.; Lu, J.; Li, R. *Coord. Chem. Rev.* 1996, **151**, 53

[4] a) Craciunescu, D.G. *et al. An. Real Acad. Farm.* 1990, **56**, 469 b) Craciunescu, D.G. *et al. An. Real Acad. Farm.* 1991, **57**, 15

[5] a) Hughes, R.G.; Bear, J.L., Kimball, A.P. *Proc. Am. Assoc. Cancer Res.* 1972, **13**, 120 b) Erck, A.; Rainen, L.; Whileyman, J.; Chang, I.M.; Kimball, A.P.; Bear, J.L. *Proc. Soc. Exp. Biol. Med.* 1974, **145**, 1278 c) Bear, J.L.; Gray, H.B.; Rainen, L.; Chang, I.M.; Howard, R.; Serio, G.; Kimball, A.P. *Cancer Chemother. Rep.* 1975, **59**, 611

[6] Kain, W.; Schwederski, B. *Bioinorganic Chemistry: Inorganic Elements in the Chemistry of Life*, John Wiley & Sons, Sussex, 1994.

[7] Boyar, E.B.; Robinson, S.D. *Coord. Chem. Rev.* 1983, **50**, 109

[8] See for example, a) Zyngier, S.B.; Kimura, E.; Najjar, R. *Braz. J. Med. Biol. Res.* 1989, **22**, 397 b) Piraino, P.; Fimiani, V.; Ainis, T.; Cavallaro, A. *J. Chemother.* 1990, **2**, 319 c) Nothenberg, M.S.; Takeda, G.K.F.; Najjar, R. *J. Inorg. Biochem.* 1991, **42(3)**, 217 d) Piraino, P.; Tresoldi, G.; Lo Schiavo, S. *Inorg. Chimica Acta* 1993, **203**, 101 e) Lo Schiavo, S.; Sinicropi, M.S.; Tresoldi, G.; Arena, C.G.; Piraino, P. *J. Chem. Soc. Dalton Trans.* 1994, **10**, 1517 f) Joesten, H.D.; Najjar, R.; Hebrank, G.P. *Polyhedron* 1982, **1(7-8)**, 637 g) Farrell, N.P.; Williamson, J.; McLaren, D.J.M. *Biochem. Pharmacol.* 1984, **33(7)**, 961 h) Souza, A.R., Ph.D. dissertation, *Rhodium (II) cycloalcanocarboxylates: synthesis, spectroscopic and thermogravimetric studies and antitumor potential evaluation* [Portuguese], Instituto de Química, Universidade de São Paulo, 1995 i) Pruchnik, F; Du_, D. *J. Inorg. Biochem.* 1996, **61**, 55 j) Pruchnik, F.; Robert, F.; Jeannin, Y.; Jeannin, S. *Inorg. Chem.* 1996, **35**, 4261 k) Souza, A.R.; Najjar, R.; Glikmanas, S.; Zyngier, S.B. *J. Inorg. Biochem.* 1996, **64**, 1 l) Souza, A.R.; Najjar, R.; Oliveira, E.; Zyngier, S.B. *Metal Based Drugs* **1997**, 4(1), 39-41

[9] Bear, J.L. *Precious Met. Proc. Int. Prec. Met. Inst. Conf., 9th* 1985, 337

[10] Rukk, N. S.; Alikberova, L.Y.; Savinkina, E.V.; Sokolova, A.S.; Stepin, B.D. *Russ. J. Inorg. Chem.* 1990, **35(2)**, 311

[11] a) Dennis, A.M.; Howard, R.A.; Lançon, D.; Kadish, K.M.; Bear, J.L. *J. Chem. Soc., Chem. Commun.* 1982, 399 b) Dennis, A.M.; Korp, J.D.; Bernal, I.; Howard, R.A.; Bear, J.L. *Inorg. Chem.* 1983, **22(10)**, 1522 c) Schelokov, R.N.; Maiorova, A.G.; Kuznetsova, G.N.; Golovaneva, I.F.; Evstaf'eva, O.N. *Russ. J. Inorg. Chem.* 1984, **29(5)**, 766 d) Ashan, M.Q.; Bernal, I.; Bear, J.L. *Inorg. Chem.* 1986, **25**, 260 e) Johnson, S.A.; Hunt, H.R.; Neumann, H.M *Inorg. Chem.* 1963, **2(5)**, 960 f) Zoroddu, M.A.; Dallochio, R. *Trans. Metal Chem.* 1989, **14(4)**, 267

[12] Clarke, M.J.; Stubbs, M. *Metal Ions Biol. Systems* 1996, **32**, 727

[13] Rempel, G.A.; Legzdins, P.; Smith, H.; Wilkinson, G. *Inorg. Synth.* 1971, **13**, 90

[14] Poizat, O.; Strommen, D.P.; Maldivi, P., Giroud-Godquin, A.M.; Marchon, J.C. *Inorg.Chem.* 1990, **29**, 4851

[15] a) Norman, J.G.; Kolari, H.J. *J. Am. Chem. Soc.* 1978, **100 (3)**, 791 b) Miskowski, V.M.; Schaefer, W.P.; Sadeghi, B.; Santarsiero, B.D.; Gray, H.B. *Inorg. Chem.* 1984, **23**, 1154 c) Martin Jr., D.S.; Webb, T.R.; Robbins, G.A.; Fanwick, P.E. *Inorg. Chem.* 1979, **18**, 475 d) Best, S.P.; Chandley, P.; Clark, R.J.H.; McCarthy, S.; Hursthouse, M.B.; Bates, P.A. *J. Chem. Soc., Dalton Trans.* 1989, 581

[16] Silverstein, R.M.; Bassler, G.C.; Morrill, T.C. *Spectrometric identification of organic compounds*, John Wiley & Sons, 5th ed, 1991.

[17] Pruchnik, F.; Hanuza, J.; Hermanowicz, K.; Wajda-Hermanowicz, K.; Pasternak, H.; Zubar, M. *Spectrochimica Acta*, 1989, **45A(8)**, 835.

[18] San Filippo, J.; Sniadoch, H.T. *Inorg. Chem.* 1973, **12**, 2326

[19] Clark, R.J.H.; Hempleman, A.J.; Flint, C.D. *J. Am. Chem. Soc.* 1986, **108**, 518

[20] Clark, R.J.H,; Hempleman, A.J. *Inorg. Chem.* 1989, **28**, 746

[21] Miskowski, V.M.; Dallinger, R.F.; Christoph, G.G.; Morris, D.E.; Spies, G.H.; Woodruff, W.H. *Inorg. Chem.* 1987, **26**, 2127

[23] a) Hydes, P.C.; Russell, M.J.H. *Cancer Metast. Rev.* 1988, **7**, 67-89 b) Cleare, M.J.; Hoeschele, J.D. *Bioinorg. Chem.* 1973, **2**,.187

**Received: October 20, 1997 - Accepted: December 3, 1997 - Received in revised camera-ready format: December 4, 1997**

